# 

**Published:** 1889-02

**Authors:** 


### EDITORIAL.

### Meeting of the Mississippi Valley Dental Society.

The annual meeting of this organization will be held, beginning March 6th and continuing three days (see official notice in this number).

It is to be hoped that this meeting will be largely attended; that not only will all the members, new and old be present, but that a large number of those who have not as yet been identified with it, may be present, and become a part of it. In the past its membership has embraced dentists from all parts of the country.

It has been the pioneer in the introduction of many phases and modes of association work, and in demonstrating their practicability and value.

By reference to the notice it will be seen that a good programme has been provided by the Executive Committee, one that will doubtless bring out much that is instructive and interesting. There are no geographical limits to this society, so then let all who can possibly reach it, come to this meeting. And not only come to see and to hear, but to be seen and to be heard.

### Annual Meeting of the Mississippi Valley Association of Dental Surgeons, Cincinnati, O., March 6th, 1889.

The forty-fifth annual meeting of the Mississippi Valley Association of Dental Surgeons will be held in Lincoln Club Hall, 8th and Race sts, Cincinnati, Ohio, on the first Wednesday in March (6th) 10 A. M. This meeting will be of especial interest to the profession.

The subjects for discussions and papers to be read are as follows:

1. Pyorrhœa Alveolaris..............H. A. SMITH, Cincinnati.
2. The Nervous Patient...........J. H. CALLAHAN, Hillsboro.
3. Digital Culture..................C. M. WRIGHT, Cincinnati.
4. Calcific Inflammation and Treatment of Pyorrhœa Alveolaris..............................J. G. TEMPLETON, Pittsburgh.
5. Voluntary Paper ...........B. O. STEVENS, Hannibal, Mo.
6. Title of Paper not Given..........WILLIAM M. MORRISON, ........................................................ St. Louis.
7. Volunteer Paper......................L. CUSTER, Dayton, O.

The principal feature of this meeting will be the great number of clinics. Drs. How, Philadelphia; Butler, Cleavland; Morrison, St. Louis; Moore, Detroit; Callahan, Hillsboro; Hinkley, Cincinnati; Barricklon, Cincinnati; Conrad, St. Louis; McMillen, Alton; and a number of others have signified a willingness to be present and clinic.

OFFICERS.

H. L. MOORE, Cincinnati, O.............................President.
J. R. CALLAHAN, Hillsboro.........................Vice-President.
W. H. FLETCHER, Cincinnati.............Second Vice-President.
F. M. SAGE, Cincinnati................Corresponding Secretary.
JAMES SILCOTT, Washington C. H...........Recording Secretary.
F. A. HUNTER, Cincinnati ..................................Treasurer.

COMMITTEES.

EXECUTIVE.—E. G. BETTY, WM. CONRAD, O. N. HEISE.
PUBLICATION.—W. M. MORRISON, L. CUSTER, WM. WILLIAMS.
MEMBERSHIP.—J. TAFT, J. I. TAYLOR.
ETHICS.—J. E. CRAVENS, J. I. TAYLOR, BINFORD.

### CONTENTS.

COMMUNICATIONS.

Dental Transplantation and Replantation.......... 57
Shedding of Temporary Teeth as to the Process.......... 65
The Physician and Dentist.......... 69
MONTHLY DIGEST..........75 and 84

PROCEEDINGS.

Odontological Society of Pennsylvania..........100

EDITORIAL.

Meeting of the Mississippi Valley Dental Society..........105

Annual Meeting of the Mississippi Valley Association of Dental Surgeons..........106

Dental Department of Meharry Medical College..........107

A New Text Book..........107

### THE DENTAL REGISTER,

A MONTHLY MAGAZINE DEVOTED TO THE INTERESTS OF THE

**DENTAL PROFESSION.**

*Terms Reduced to* $2.00 *Per Year. Single Copies,* 25 *Cents.*

**EDITED BY PROF. J. TAFT, D.D.S.**

—AND—

**Published by SAM'L A. CROCKER & CO., Ohio Dental and Surgical Depot.**

The volume commences in January and closes in December.

The REGISTER being issued monthly is always fresh, therefore Indispensable to the practitioner who desires to keep posted up with the new inventions and improvements which are daily developed. Neither expense nor effort will be spared to give value and variety to its contents. Articles will be illustrated with well executed engravings whenever they will add to the interest or assist to explanation.

Its extensive circulation and frequency of issue make it one of the BEST DENTAL ADVERTISING MEDIUMS in the United States.

All subscriptions must begin with January or July.

Local or special notices, five lines or less, will be inserted for $3 each insertion; over five lines, 50 cts. per line.

All advertising bills payable in advance, or at furthest, after the issue of the first number containing the advertisement. All transient advertisements to be paid for in advance.

☞CONTRIBUTIONS SOLICITED.

Exclusively Editorial Communications should be addressed to the Editor.

The latest number of the REGISTER may be ordered with or without other goods at any time, and all letters should be addressed to

**SAM'L A. CROCKER & CO., Ohio Dental and Surgical Depot, Cincinnati, O.**

